# Cannabinoid_1_ (CB-1) receptor antagonists: a molecular approach to treating acute cannabinoid overdose

**DOI:** 10.1007/s00702-019-02132-7

**Published:** 2019-12-31

**Authors:** Phil Skolnick, Roger Crystal

**Affiliations:** Opiant Pharmaceuticals, Inc., 233 Wilshire Boulevard, Suite 280, Santa Monica, CA 90401 USA

**Keywords:** Cannabinoid_1_ receptors, Tetrahydrocannabinol, Synthetic cannabinoids, Cannabinoid antagonists, Acute cannabinoid overdose, Cannabinoid intoxication

## Abstract

The legalization of cannabis for both recreational and medical use in the USA has resulted in a dramatic increase in the number of emergency department visits and hospital admissions for acute cannabinoid overdose (also referred to as cannabis intoxication and cannabis poisoning). Both “edibles” (often sold as brownies, cookies, and candies) containing large amounts of Δ^9^-tetrahydrocannabinol and synthetic cannabinoids (many possessing higher potencies and efficacies than Δ^9^-tetrahydrocannabinol) are responsible for a disproportionate number of emergency department visits relative to smoked cannabis. Symptoms of acute cannabinoid overdose range from extreme lethargy, ataxia, and generalized psychomotor impairment to feelings of panic and anxiety, agitation, hallucinations, and psychosis. Treatment of acute cannabinoid overdose is currently supportive and symptom driven. Converging lines of evidence indicating many of the symptoms which can precipitate an emergency department visit are mediated through activation of cannabinoid_1_ receptors. Here, we review the evidence that cannabinoid_1_ receptor antagonists, originally developed for indications ranging from obesity to smoking cessation and schizophrenia, provide a molecular approach to treating acute cannabinoid overdose.

## Introduction

In 2012, Colorado and Washington legalized the recreational use of cannabis. Over the past 7 years, nine more states and Washington, DC, have followed suit; about 25% of the US population now lives in states where it is legal to produce and sell cannabis to adults. In addition, fifteen states have decriminalized possession of small quantities of cannabis (ranging from 10–100 g), and thirty-three states have legalized medical use. Ironically, state laws that either legalize or decriminalize cannabis are in violation of US Federal law prohibiting the growing, use, and sale of cannabis and cannabis containing products. Both the rapidly changing legal landscape and the growing perception that there is little harm in cannabis use (Aubrey 2019; Volkow, et al., 2014) have resulted in a dramatic increase in cannabinoid-related emergency department (ED) visits. Thus, in 2011 there were about 456,000 cannabis-related ED visits in the USA (Volkow et al. 2014; Richards et al. 2017a, b). Based on a recent analysis of the National Emergency Department Sample, we estimate there will be in excess of 1.7 million cannabis-related ED visits in 2019. There are currently no approved medications to reverse symptoms resulting from an acute cannabinoid overdose (ACO) which can trigger an ED visit (Zaurova et al. 2016), and in more severe cases, hospitalization (Winstock et al. 2015). Terms like cannabis poisoning and marijuana intoxication have also been used to describe ACO (Kim and Monte 2016; Ishak et al. 2018); there are multiple International Classification of Diseases (ICD) codes using descriptors such as cannabis intoxication and cannabis poisoning (Monte et al. 2019). Current treatment is supportive and symptom driven (Hermanns-Clausen et al. 2013; Winstock et al. 2015; Kim and Monte 2016; Cooper 2016). Given the likelihood that cannabis-related ED visits and hospitalizations will continue to increase as states continue to either legalize or decriminalize cannabis use, developing therapeutics that can specifically mitigate these symptoms represent a meaningful advance in treatment that could reduce the time spent in EDs, decrease the number of ED visits that result in hospitalization, and reduce the length of stay should hospitalization be required.

Converging lines of evidence, including compelling clinical data, have shown that cannabinoid (CB-1) receptors mediate the principal psychopharmacological actions of both Δ^9^-tetrahydrocannabinol (THC) (the main psychoactive component in cannabis) and synthetic cannabinoids (SCs) that are likely to precipitate an ED visit (Huestis, et al. 2001; Baskfield et al 2004; Zimmer et al. 1999; Sain et al. 2009; Pryce and Baker 2017; Marshell et al 2014; Zuurman et al. 2010; Klumpers et al. 2012). The identification of CB-1 receptors in 1990 (Matsuda et al. 1990) and an emerging literature describing the role of endocannabinoids (e.g., anandamide) in multiple physiological processes (reviewed in Howlett et al. 2002; Pertwee et al. 2010) led to the development of selective, high affinity CB-1 receptor antagonists. A handful of these molecules entered clinical trials in indications ranging from schizophrenia to obesity (LeFoll et al. 2009; Janero and Makriyannis 2009). The CB-1 antagonist, rimonabant (Acomplia®, Sanofi), was approved for the treatment of obesity in more than 50 countries and was also in development for smoking cessation (Lefoll et al. 2009). However, concerns about the psychiatric side effects (notably increased rates of anxiety, depression, and suicidal ideation) of rimonabant that emerged with chronic administration (Moreira et al. 2009) led the EMEA to suspend marketing in late 2008. Sanofi quickly withdrew rimonabant from the market and halted further development of CB-1 antagonists. This led to the rapid termination of competing programs by Pfizer, Merck, Astra-Zeneca, and others, with several of these molecules in mid-to-late stage clinical development. In this review, we describe both the characteristics of ACO and the evidence that high affinity CB-1 antagonists can be employed to treat the symptoms of ACO through a receptor-mediated reversal of the pharmacological actions of both THC and SCs.

### Scope of the problem

Marijuana is the most commonly used illicit drug in the USA. Between 2010 and 2016, the number of individuals using marijuana in the past month increased by ~ 30%, from approximately 25 million to 32 million (Midgette et al. 2019). The increased number of individuals using marijuana and other cannabis-derived products on a regular basis is just one factor contributing to the dramatic rise in the number of cannabis-related ED visits. Thus, both the availability of very high content THC products (THC concentrations of 50% have been found in hash oil products used in “dabbing”; high end recreational and medical flower cannabis approaches 30% THC compared to a “historical” 4–8% in flower cannabis] (Al-Zouabi et al. 2018) and increasingly permissive attitudes toward use in both the recreational and medical settings have also contributed to this problem. While the use and potency of THC-containing products continue to increase, the rapid absorption of THC from smoked and vaporized cannabis (peak blood concentrations can be achieved within 10 min) and the onset of intoxicating effects which parallel absorption from the lungs (Spindle et al. 2018) tend to limit an individual’s THC intake. High THC content edible products (“edibles”) (Monte et al 2019) and synthetic cannabinoids (SCs) now account for a disproportionate number of cannabinoid-related ED visits and hospital admissions relative to smoked cannabis (Monte et al 2019; Winstock et al. 2015).

a) “Edibles”: Edible products represent a small fraction of total cannabis sales, but account for a disproportionate percentage of cannabis-related ED visits. For example, in the bellwether state of Colorado (recreational use of cannabis was legalized in 2012), edibles accounted for ~ 0.32% used (on the basis of THC content) between 2014–2016, but were responsible for ~ 10.7% of cannabis-related ED visits during this period (Monte et al. 2019). Edibles produced a higher incidence of intoxication (48% versus 28%), psychiatric (18% versus 8.4%), and cardiovascular (8% versus 3.1%) symptoms compared to smoked cannabis (Monte et al. 2019). Edibles are packaged in foods and drinks (e.g., brownies, cookies, chocolates, fruit snacks, popcorn, and sodas) containing very high quantities of Δ^9^-tetrahydrocannabinol (THC) relative to smoked cannabis. For example, Vandrey et al. (2017) reported a median dose of 54 mg THC found in 75 edible products in cannabis dispensaries in California and Washington State; an “average joint” has been estimated to contain 7–8 mg THC (Kögel et al. 2017). There is 10 mg of THC in a standard edible “dose” [e.g., the amount in a small chocolate drop or fruit slice (Denver Public Health 2019)], but THC-containing products that can be purchased on the internet advertise products containing 100–1000 mg of THC in, for example, a brownie or candy bar intended to be divided into multiple “doses.” The high THC content of these products often leads to unintentional overconsumption, and the slow absorption delays onset (measured, for example, by VAS high) 0.5–2 h (Vandrey et al. 2017) compared to smoked or vaporized cannabis (Spindle et al. 2018). This delay increases the potential for consumption of additional quantities because of the perception that the edible is “not working” (Dowd 2014). Children are particularly vulnerable to overdose with high content THC edibles often resembling treats (Richards et al. 2017a, b; Cao et al. 2016). In Colorado, the number of cannabis-related hospital visits doubled for children under 9 in the 2 years after recreational cannabis was legalized compared to the prior 2 years (Wang et al. 2016); approximately 35% of pediatric cases presenting to the hospital require admission (Wang et al. 2016).

b) Synthetic cannabinoids (SCs): Following the identification of CB-1 receptors (Matsuda et al. 1990), both industry and academia synthesized specific, high affinity agonist and antagonist ligands to explore receptor biology and as potential therapeutics. Hundreds of these compounds have been reported in the peer reviewed literature (reviewed in Castaneto et al. 2014). SCs are structurally diverse agonists, and many of these compounds possess significantly higher affinities than THC at CB-1 receptors (Table 1). THC is reported to act as a partial agonist at CB-1 receptors in functional assays, and multiple SCs have also been reported to possess significantly higher efficacies than THC (Burkey, et al. 1997; Castaneto et al. 2014). For example, using stimulation of [^35^S]GTPγS binding to mouse brain membranes as a functional efficacy measure, Burkey et al. (1997) reported that CP 55,940 and HU 210 (Table 1) were four- and twofold more efficacious than THC, respectively.

**Table 1:** Affinities of synthetic cannabinoids for CB-1 receptors: comparison with THC

Compound	Chemical class	K_i_ (nM)	THC/SC
AB-FUBINACA	Indazole carboxamide	0.9	45.6
AM694	Benzoylindoles	0.1	410
AM1220	Naphthoylindoles	3.9	10.5
AM1248	Adamantylindoles	11.9	3.4
( ±) CP47,497	Cyclohexylphenols	2.2 ± 0.5	18.6
CP55,940	Cyclohexylphenols	1.1 ± 0.04	37.3
HU-210	Dibenzopyran	0.2	205
JWH-018	Naphthoylindoles	9.0 ± 5.0	4.6
JWH-250	Phenylacetylindoles	11.0 ± 2.0	3.7
JWH-307	Naphthoylpyrroles	7.7	5.3
THC	Dibenzoypyran	41 ± 2	1.0
WIN55,212–2	Aminoalkylindoles	62.3	0.7

These compounds are representative of the molecules described in the patent, chemical, and biological literature, and illustrate both the structural diversity and potency of synthetic cannabinoids compared to THC, the principal psychoactive molecule in cannabis. These data are from Castaneto et al. (2014)

SCs are most often dissolved in an organic solvent such as acetone and sprayed on herbs such as oregano or other plant materials. These products, sometimes collectively referred to as “Spice,” are sold under names such as K2, Purple Haze, and Flying Buddah (Castaneto et al. 2014; Cooper 2016). Despite attempts by the US Drug Enforcement Administration to restrict distribution by scheduling many SCs as “Class I” substances, the ability to make small chemical modifications to multiple core structures (Table 1) that bind to CB-1 receptors with high affinity is problematic from an enforcement standpoint. Often marketed as herbal incense and labeled “not for human consumption” to avoid regulation, SCs are sold in gas stations, “head” shops, and through the internet. SC-containing products are unregulated, and hence, the quantity, identity, and purity of compound(s) contained in these products can vary widely. SCs are attractive to users because they are less expensive than THC-containing products and generally not detected by routine drug screens. While SC use is not widespread relative to cannabis, the relative risk of an individual seeking emergency medical treatment has been estimated to be 30-fold higher (Winstock et al. 2015) for SC users, with the potential for multiple victims to severely tax the resources of most EDs (Winsock and Jacobo, 2018).

c) Symptoms: Symptoms produced by both cannabis products and SCs that can precipitate an ED visit include extreme lethargy, ataxia, decreased concentration and generalized psychomotor impairment, feelings of panic and anxiety, agitation, delirium, hallucinations, psychosis, tachycardia, and nausea/vomiting. Symptom severity varies with the route of administration, quantities used, as well as the age of the patient (Castaneto et al. 2014; Monte et al. 2017; Winstock et al. 2015; Kim and Monte 2016; Zaurova et al. 2016). Hermanns-Clausen et al. (2013) report that the adverse events produced by SCs were mostly similar to those produced by high dose cannabis (i.e., THC-containing products). However, both the higher potencies and efficacies of SCs compared to THC (Castaneto et al. 2014; Burkey et al. 1997) may be responsible, at least in part, for the severity of side effects producing a much higher incidence of ED visits (Winstock et al. 2015). While speculative, it is these pharmacodynamic properties that may also produce symptoms generally viewed as unique to SCs including seizures, hypertension, and hypokalemia (Hermanns-Clausen et al. 2013); other reported symptoms including cardiac arrest, nephrotoxicity, and severe rhabdomyolysis (Cooper 2016) could be related to contaminants (e.g., chemical intermediates, solvents, and other materials added during processing and product preparation) in SC-containing products rather than CB-1 receptor-mediated actions.

Symptoms were reported to resolve within 6–12 h in about half of patients, but the literature is replete with reports that symptoms produced by both edibles and SCs can persist for significantly longer, lasting from days to several weeks in some patients (Hermanns-Clausen et al. 2013; Hudak et al. 2015; Bui et al. 2015; Winstock et al. 2015).

### Can CB-1 antagonists be used to treat ACO?

While not originally envisioned as an emergency treatment for ACO, converging lines of evidence have demonstrated that competitive CB-1 receptor antagonists can both block and reverse the pharmacological actions of THC and SCs. For example, both THC and SCs produce a well-described pharmacology in rodents, commonly referred to as the cannabinoid tetrad. These effects (hypothermia, analgesia, suppression of locomotor activity, and catalepsy) can be blocked by pretreatment with CB-1 antagonists including rimonabant, surinabant, and AM 251 (Rinaldi-Carmona et al., 1994; 2004; Varvel et al. 2005; McMahon and Koek 2007; Marshell et al. 2014). For example, Rinaldi-Carmona et al. (1994) described a dose-dependent blockade of the cannabinoid tetrad produced by intravenous injection of the SC, WIN55212-2 by the prototypic CB-1 antagonist, rimonabant. Consistent with these findings, rimonabant blocked the cannabinoid tetrad produced by the SCs JWH-018 and JWH-073 administered by either injection or inhalation (Marshell et al. 2014). Similarly, Rinaldi-Carmona et al. (2014) demonstrated that surinabant blocked multiple pharmacological actions of the SCs CP55,940 and WIN55212-2, including hypothermia and reduced gastrointestinal transit.

Multiple studies (e.g., Varvel et al. 2005; McMahon and Koek 2007; Marshell et al. 2014) have also demonstrated that rimonabant blocks the hypothermic, antinociceptive, and cataleptic effects of both parenterally administered and inhaled THC. Varvel et al. (2005) reported this blockade was effective against THC as well as an ethanolic extract of marijuana (containing cannabinoids other than THC) adulterated with varying amounts of THC. McMahon and Koek (2007) reported a striking similar behavioral profile of rimonabant and AM 251 to block the hypothermia and catalepsy and partially attenuate the hypoactivity produced both THC and WIN555212-2. This description is intended to provide the reader with a very brief overview of a preclinical literature replete with reports demonstrating that structurally diverse CB-1 antagonists (Janero and Makriyannis 2009) can block the pharmacological actions of both SCs and THC.

**Fig. 1:**
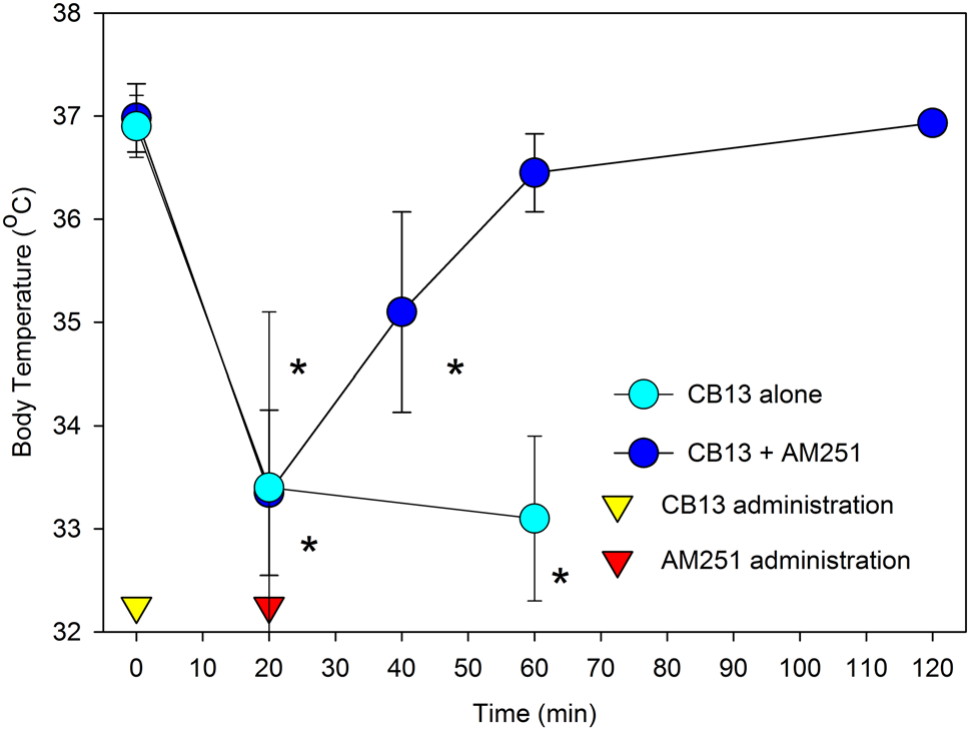
The CB-1 receptor antagonist AM251 reverses the hypothermia produced by CB-13, a CB-1 receptor agonist. Mice were injected with CB-13 (5 mg/kg, i.p.) and AM251 (5 mg/kg, i.v.) administered 20 min. later. Data represent group means ± SD. *p < 0.05 compared to baseline values. Light circles: CB-13; dark circles: CB-13 + AM 251; open triangle: administration of CB-13 (time zero); closed triangle administration of AM251 (time: 20 min.). The figure is reprinted from G. Pryce and D. Baker (2017) Brit. J. Pharmacol. 174:3790–3794, with permission of the authors

CB-1 antagonists have also been shown to reverse the pharmacological effects of cannabinoids. For example, the CB-1 antagonist AM-251 has been reported to reverse the hypothermia induced by the SC, CB13 (Pryce and Baker 2017). In this study (Fig. 1), parenteral administration of CB13 produced a significant (~ 4 ^o^C) drop in core temperature within 20 min. which was maintained for at least 60 min. Intravenous administration of AM-251 partially reversed this hypothermia within 20 min., and fully reversed it within 40 min. Using a hippocampal slice preparation, Hoffman et al. (2016) demonstrated that the CB-1 antagonists AM-251 and PIMSRI1 rapidly reversed the inhibition of synaptic transmission produced by both THC and SCs. These latter findings are particularly relevant from a translational perspective because CB-1 receptor activation is known to inhibit transmitter release (in this instance, glutamate) from axon terminals. The well-described cognitive impairment produced by cannabinoids may well be a reflection of this phenomenon (Sullivan 2000).

While fully consistent with the principle of mass action, the demonstration that CB-1 antagonists can both block and reverse the pharmacological effects of cannabinoids (both THC and SCs) in vitro and in vivo de-risks the clinical translation of using a CB-1 antagonist as a reversal agent. Moreover, in addition to blocking the cannabinoid tetrad, CB-1 antagonists have also been shown to block more complex cannabinoid-induced behaviors. For example, using non-human primates trained to administer THC intravenously, Justinova et al. (2008) reported that rimonabant blocks both the direct reinforcing effects of THC and THC-induced drug seeking. Again, the studies cited here are not meant to provide an exhaustive review of this literature, but rather provide the reader with the perspective that a large preclinical literature is consistent with the hypothesis that CB-1 antagonists can be used to treat ACO.

Perhaps even more compelling than this preclinical literature are multiple clinical reports, demonstrating that CB-1 antagonists block both the objective and subjective effects of inhaled THC and smoked marijuana. Huestis et al. (2001) first reported that 90 mg of oral rimonabant administered 2 h prior to smoking a marijuana cigarette (with an average weight of 764 mg and 2.64% THC content, containing about 20 mg THC) reduced both subjective intoxication and tachycardia. In this study, subjects also smoked cigarettes (THC was removed by solvent extraction) that were identical in appearance and smell in order to appropriately control for placebo effects. Rimonabant significantly reduced visual analog scale (VAS) scores to questions such as “How high do you feel now?” and “How strong is the drug effect you feel now?” by 38–43%, and reduced drug induced elevations in heart rate by 59%. These effects of rimonabant persisted for the duration of the observation period (60–65 min. after initiating smoking). The metabolism of THC appeared unchanged by rimonabant since neither peak plasma THC levels nor area under the curve measurements (up to 1.8 h after smoking) was affected.

Two subsequent proof of principle studies using chemically distinct CB-1 antagonists and vaporized THC (rather than smoked marijuana) are consistent with the hypothesis that this class of compound will effectively reverse the symptoms of ACO. Zuurman et al. (2010) demonstrated that drinabant (AVE1625), which had been evaluated in multiple Phase I and Phase II studies (Janero and Makryiannis 2009), blocked both the objective and subjective effects of inhaled THC. In this double blind, placebo-controlled study, healthy male subjects (who were experienced, but not chronic cannabis users) received oral drinabant doses of 20, 60, or 120 mg. Three hours later, subjects inhaled a total of 18 mg THC (or placebo) vapor over 4 h: 2 mg initially, followed by doses of 4, 6, and 6 mg at one, two, and three hours later. THC-induced changes in objective measures, including heart rate and body sway were substantially reduced at all doses of drinabant. For example, the peak effect of THC-induced tachycardia was inhibited between 89 and 109%, while body sway was inhibited between 61 and 74%, respectively. Moreover, subjective measures such as THC-induced changes in (rated using a VAS scale) alertness, feeling “high,” and measures of internal and external perception were also reduced at all doses of drinabant (Table 2; Zuurman et al. 2010). The latter two measures merit additional comment: external perception reflects a subject’s misperception of external stimuli or a change in awareness of the current surroundings. This score is calculated as an average of VAS scores that include: altered passage of time, changing of body parts, changes in sound intensity, and changes in color intensity. Internal perception reflects inner feelings that do not correspond with reality such as feelings of unreality, hearing sounds/voices, paranoia, and feeling anxious. It is likely that the distress precipitating many ED visits is related to THC-induced alterations in both internal and external perception. Thus, the ability of a CB-1 antagonist to block these effects is consistent with the hypothesis that rapid delivery of a CB-1 antagonist in an ED setting should result in a reversal of these symptoms.

**Table 2:** Blockade of THC-induced subjective and objective measures by drinabant

Measure	Inhibition ratios (with 95% CI) at THC peak effect		
	20 mg Drinabant	60 mg Drinabant	120 mg Drinabant
Heart rate	89% (61, 118)	96% (66, 126)	109% (78, 140)
Body sway	61% (22, 100)	73% (32, 113)	74% (33, 114)
VAS alertness	61% (25, 97)	76% (37, 114)	94% (52, 136)
VAS “feeling high”	90% (72, 107)	83% (66, 99)	101% (83, 120)
Internal perception	103% (62, 144)	86% (49, 122)	71% (37, 105)
External perception	83% (60, 105)	90% (67, 113)	88% (65, 111)

Inhibition ratios are calculated with the following formula: [(drinabant + THC)—(placebo drinabant + THC)] / [(placebo drinabant + THC vehicle)—(placebo drinabant + THC)]. The data in this table are from Zuurman et al. (2010)

There were differences in both the sensitivity of objective and subjective measures to THC and as well as their duration. For example, THC-induced increases in heart rate were not remarkable until a total of 12 mg of vapor had been inhaled, and returned to placebo values ~ 2 h after the last dose of THC. In contrast, VAS changes in feeling “high” were increased after the first dose 2 mg dose of THC, peaked at 12 mg of THC, and remained above placebo levels for at least 7 h (Zuurman et al. 2010). The effects of drinabant were sustained for the entire observation period, that is, for 9 h after the initial dose of THC. Despite dose-dependent increases in plasma drinabant concentrations, there was no compelling evidence of dose dependence for most of these measures (Table 2 and Zuurman et al. 2010).

Klumpers et al. (2012) used an identical study design (including dosing schedule and endpoints) to demonstrate that pretreatment (1.5 h) with the CB-1 antagonist surinabant (20 and 60 mg orally) dramatically reduced THC-induced changes in body sway, heart rate, and multiple subjective measures including VAS alertness, feeling high, and both internal and external perception. Only feeling high and heart rate were not judged to be completely blocked at either dose of surinabant, but peaked at around 70% for both measures (Klumpers et al. 2012). This study noted that the 60 mg dose of surinabant had no effects on mood. In this study, the authors made population PK-PD parameter estimates for body sway and multiple subjective measures. Plasma IC_50_ values for surinabant ranged from 22 ng/ml for blocking body sway to 58.8 ng/ml for blocking changes in internal perception, respectively, although it is noted that the relative standard errors for these estimates were large (Klumpers et al. 2012). Nonetheless, based on based on these values, the authors concluded that plasma concentrations following 20 mg orally appear maximum for blocking these effects of intrapulmonary THC. The most likely explanation for the apparent lack of dose dependence by both molecules is that plasma concentrations of drinabant and surinabant following oral administration of as little as 20 mg were sufficient to produce a sustained reduction of the pharmacological actions produced by 18 mg of inhaled THC. The high affinities of drinabant (Bertalovitz et al. 2010) and surinabant (Rinaldi-Carmona et al. 2004) at CB-1 receptors relative to THC (Burkey et al. 1997; Castaneto et al. 2014) are consistent with this hypothesis. Both the studies of Zuurman et al. (2010) and the Klumpers et al. (2012) measured drug plasma concentrations over time, but there are too many variables, ranging from oral bioavailability to plasma protein binding and brain penetration, that preclude a meaningful comparison of relative in vivo potencies between these two molecules.

## Conclusions

The use of a molecular approach to reverse the symptoms of ACO is well grounded in the principles of pharmacology. Thus, the strategy of using a high affinity, specific receptor antagonist to reverse the pharmacological actions of an agonist ligand has successfully employed in the treatment of both opioid and benzodiazepine overdose.

Rimonabant was withdrawn from the market based on reports of anxiety, depression, and suicidal ideation (Moreira et al. 2009; LeFoll et al. 2009) in long-term obesity and overweight trials (e.g., Rucker et al. 2007). In retrospect, it is perhaps unsurprising that chronic, high affinity blockade of CB-1 receptors could manifest these symptoms in some individuals if the psychopharmacological actions of THC mirror, at least to some extent, the physiological functions of endocannabinoids. However, neither single doses nor short-term administration of CB-1 antagonists produces these adverse events (Huestis et al. 2001, 2007; Zuurman et al. 2010; Klumpers et al. 2012).

Several CB-1 antagonists, including rimonabant and AM-251, have been reported to possess inverse agonist properties (Pertwee 2005), and it has been hypothesized these inverse agonist properties may contribute to the emergence of psychiatric side effects in some patients following long-term treatment with rimonabant (LeFoll et al. 2009; Moreira et al. 2009; Porcu et al., 2018). Nonetheless, at a cellular level, CB-1 antagonists that do not appear to possess inverse agonist properties (sometimes called “neutral” antagonists) can both block and reverse the effects of exogenously applied cannabinoids (Hoffman et al. 2016). These data indicate that an inverse agonist action is not necessary for an effective reversal agent. Further, while rimonabant binds to CB-1 receptors with an affinity of ~ 2 nM (Rinaldi-Carmona et al., 1994), in many in vitro model systems, inverse agonist actions are manifested only at much higher (low μM) concentrations (Pertwee 2005; Porcu et al. 2018) that would not be encountered following a single administration of rimonabant (or another high affinity CB-1 antagonist) used as a rescue agent. Thus, both preclinical and clinical data indicate that a high affinity, specific CB-1 antagonist offers a highly favorable risk/benefit profile for single use as a reversal agent for the treatment of ACO. While oral administration is impractical both because of a slow onset (e.g., Zuurman et al 2010; Klumpers et al. 2012) and patients who may be unable (e.g., intoxicated) or unwilling (agitated) to take an oral medication, reformulation of a CB-1 antagonist for parenteral administration offers the potential for a more rapid onset and ease of delivery in an ED setting.
